# Correlates of Unmet Need for Modern Contraception Among Reproductive-Aged Women Involved in New York City Criminal Legal Systems

**DOI:** 10.1089/whr.2023.0177

**Published:** 2024-02-16

**Authors:** Melissa N. Slavin, Brooke S. West, Deanna Schreiber-Gregory, Frances R. Levin, Gina Wingood, Steve Martino, Golfo Tzilos Wernette, Chermaine Black, Nabila El-Bassel

**Affiliations:** ^1^ School of Psychology and Counseling, Fairleigh Dickinson University, Teaneck, New Jersey, USA.; ^2^ Social Intervention Group, Columbia School Social Work, Columbia University, New York, New York, USA.; ^3^ Juxdapose, LLC, Gaithersburg, Maryland, USA.; ^4^ Department of Psychiatry, Columbia University Irving Medical Center/New York State Psychiatric Institute, Columbia University, New York, New York, USA.; ^5^ Department of Sociomedical Sciences, Mailman School of Public Health, Columbia University, New York, New York, USA.; ^6^ Department of Psychiatry, Yale School of Medicine, New Haven, Connecticut, USA.; ^7^ Department of Family Medicine, University of Michigan Medical School, Ann Arbor, Michigan, USA.

**Keywords:** unmet need, contraception, intimate partner violence, substance use, criminal justice, mental disorders

## Abstract

**Introduction:**

The population of women involved in criminal legal systems (WICL), a majority of whom are reproductive-aged, has risen steadily in the United States. They contend with numerous barriers to sexual and reproductive health services resulting in high rates of unmet need for contraception and unintended pregnancy.

**Materials and Methods:**

This study included 132 non-pregnancy seeking reproductive-aged WICL enrolled in the baseline assessment of the HIV prevention intervention, “Women on the Road to Health” (WORTH). A multivariate generalized linear logistic regression model with robust estimation examined effects of past 6-month intimate partner violence (IPV; sexual and physical/injurious), past 3-month substance use (binge drinking, cannabis, other illegal drug use), and lifetime mental health diagnoses (anxiety, depression, bipolar disorder) on women's unmet need for modern contraception, adjusting for significant demographic and socioeconomic factors.

**Results:**

Women who were younger in age (odds ratio [OR]: 0.74; 95% confidence interval [CI]: 0.63–0.88) and reporting lifetime diagnoses of anxiety disorders (OR: 13.64; 95% CI: 2.71–68.34) were significantly more likely to meet the criteria for unmet need for modern contraception. Women with a regular gynecologist (OR: 0.11; 95% CI: 0.01–0.86) reporting lifetime diagnoses of bipolar disorder and past 6-month sexual IPV histories (OR: 0.04; 95% CI: 0.002–0.86) were significantly less likely to meet the criteria for unmet need for modern contraception.

**Conclusions:**

Distinct mental health diagnoses and experiences of IPV may uniquely impact unmet need for modern contraception among WICL. These findings emphasize the need for a more nuanced comprehension of these relationships to deliver comprehensive and holistic health services that address the intersecting needs of this population.

Trial registration: ClinicalTrials.gov NCT01784809. Registered 6 February 2013.

## Introduction

The population of women involved in criminal legal systems (WICL) in the United States has surged by 525% between 1980 and 2021, primarily due to drug-related offenses stemming from the “war on drugs.”^1^ A majority of WICL are reproductive-aged^2^ and face high rates of unmet need for contraception,^3–6^ defined by the World Health Organization (WHO) as able to conceive, sexually active, and not using contraception despite not desiring more children or wanting to delay the next child.^7^ WICL encounter barriers to sexual and reproductive health (SRH) services at the individual, interpersonal, and community levels, including high rates of substance use disorders (SUDs),^8^ mental health issues,^9^ intimate partner violence (IPV),^10,11^ and limited access to health care systems.^6,12–14^

Unmet need for contraception results in an increased prevalence of unintended pregnancies (encompassing both unwanted or mistimed pregnancies)^15^ among WICL.^6^ Women facing unintended pregnancy frequently encounter delayed pregnancy recognition, decreased engagement with prenatal care,^16,17^ higher rates of obstetric complications,^18^ increased incidents of postpartum depression and stress,^19^ and higher likelihood of premature births.^19^ These risks are further heightened among Black women who are overrepresented in the criminal legal (CL) systems^20^ and face higher rates of pregnancy-related morbidity and mortality due to structural racism.^21^ Conversely, research suggests that the availability of SRH services, including contraceptive options consistent with women's family planning needs, empowers women to make informed choices about parenthood, improving health behaviors, birth outcomes, as well as women's social and financial well-being.^22,23^

WICL may encounter additional obstacles when accessing “modern” contraception, defined as “products or medical procedures that interfere with reproduction resulting from acts of sexual intercourse,”^24^ as many of these methods, including hormonal contraceptives, necessitate prescriptions. While the term “modern contraception” aligns with the contemporary description of such practices within the field, there exist concerns surrounding its usage. Dr. James Marion Sims, recognized as the “father of modern gynecology,” conducted experiments on Black enslaved women, sparking major ethical controversies.^25^ Moreover, the term fails to acknowledge “non-modern” contraception forms such as lactational amenorrhea or fertility awareness methods, which, although effective for pregnancy prevention, require women to experience pregnancy or adjust the timing of intercourse for efficacy. In addition, the term encompasses a wide range of contraceptives with varying effectiveness, ranging from condoms to surgical sterilization procedures.^26^ Despite these complexities, due to the absence of more suitable terminology and for the sake of clarity, the article will employ the term “modern contraception.”

The inaccessibility of contraception for certain groups in Western countries, such as WICL, has become increasingly evident *via* recent studies.^3,4,6,27–29^ Research has largely focused on socioeconomic and demographic determinants, indicating that women who identify as Black or African American, with less than a high school degree, who are below the poverty line, unemployed, underinsured/uninsured, and on Medicare/Medicaid have greater unmet need for contraception.^27,30,31^ This literature points to the need for reduced costs and increased availability to contraceptive services among socioeconomically disadvantaged and minoritized women, as well as policies and systems-level interventions targeting institutional discrimination and racism.

While structural-level changes are vital for reducing unmet need for contraception among WICL, there has been comparatively less focus on synergistically connected interpersonal- and individual-level barriers. Specifically, WICL are disproportionately impacted by IPV, with more than one third of women in US state prisons disclosing physical or sexual IPV.^32^ Histories of trauma, socioeconomic disparities, and systemic inequalities can render WICL more susceptible to abusive relationships.^33^ Moreover, the intersection of CL involvement and IPV can create a feedback loop, wherein experiences of violence can contribute to subsequent encounters with the legal system.^34–36^ IPV can disempower women, reduce self-efficacy, and limit women's ability and motivation to take control of their reproductive health and access or utilize contraception.^37,38^ Furthermore, women may experience reproductive coercion, a specific type of IPV that directly interferes with their reproductive autonomy.^39–42^

Research has also documented a high prevalence of mental disorders and SUDs among WICL, influenced by histories of trauma and victimization, adverse developmental experiences, current social circumstances including homelessness and unemployment, as well as stressors associated with incarceration itself.^9,43^ National data have revealed that ∼69% of women in state prisons reported a history of a mental health problem.^44^ SUDs are particularly prevalent, occurring at almost nine times the rate among incarcerated women than the general population of women.^2,45–47^ WICL experiencing SUDs and other mental disorders encounter numerous barriers when seeking SRH care. These obstacles include financial constraints, challenges in accessing services, uncertainties pertaining to medication interactions, stigma from health care providers, as well as risks and concerns of coerced treatment, potential criminalization, and losing custody of one's children.^48–50^

To the authors' knowledge, no known study has directly examined the effects of IPV, mental health disorders, and substance use on WICL's unmet need for contraception. Research that has examined the influence of these factors have found that they frequently hinder effective contraceptive use,^3,37,51–55^ although much of this literature has not considered pregnancy risk or intentions among these samples, and have not focused exclusively on WICL.

Furthermore, there is a need to differentiate the impact between various forms of IPV (*e.g*., sexual vs. physical), specific mental health diagnoses (*e.g*., anxiety, bipolar disorder, depression), and different substances (*e.g*., alcohol, cannabis, other illegal drugs). This current study examined whether these factors predicted unmet need for modern contraception among a sample of 132 non-pregnancy seeking reproductive-aged women under community supervision. In addition, this study explored whether each significant predictor of unmet need for modern contraception was significantly associated with condom use or alternative forms of contraception.

## Methods

Data from this study came from the baseline assessment of the HIV prevention intervention, “Women on the Road to Health” (“WORTH”),^56^ which included 306 women under community supervision (probation, parole, or alternatives to incarceration). The current analysis included 132 reproductive-aged women (18–44 years) who indicated that they were not currently trying to get pregnant. To participate in the original intervention study, women must have been (1) 18 years or older and biologically female at birth; (2) under community supervision, on probation or parole, or under drug treatment court supervision within the past 90 days; (3) report one or more incidents of illegal drug use within the past 6 months; (4) report one or more acts of unprotected intercourse within the past 3 months; and (5) HIV positive or at risk for HIV.

In the original study, research staff administered surveys in-person at baseline, 3-, 6-, and 12-month follow-up periods, with biological testing conducted at baseline and 12-month follow-up. Study participants completed written informed consent for participation and were compensated with $30 for completing the baseline computer-assisted self-interview (CASI) and biological testing. All consent forms clearly stated that participation in the study was completely voluntary and would not affect women's status within the CL system. Study procedures were approved by institutional review boards at Columbia University and the Center for Court Innovation.

### Measures

#### Primary outcome: unmet need for modern contraception

The evaluation of unmet need for modern contraception involved criteria outlined in prior literature:^27^ women were considered eligible if they were able to conceive, engaged in unprotected vaginal intercourse, expressed a desire to delay or prevent pregnancy, and refrained from using any form of contraception. To gauge the likelihood of pregnancy, this study exclusively focused on women aged between 18 and 44 years. To assess lack of pregnancy intentions, only participants who answered “no” to the query, “Are you currently trying to get pregnant?” were included in this study. To examine contraceptive use, women were inquired on the number of instances of vaginal intercourse with a male partner without using a condom within the past 3 months.

In addition, questions were asked about the current usage of alternative modern contraceptive methods, including tubal ligation, hysterectomy, oophorectomy, partner's vasectomy, oral contraceptives, or intrauterine devices (IUDs). Some participants also mentioned the use of injectable contraception, implants, or transdermal patches in open-ended responses. Women were classified as having an unmet need for contraception if they reported past 3-month sexual intercourse without a condom and were not presently using any other method of contraception.

#### Secondary outcome: contraceptive type

Individual instances of condomless sex (continuous) and other contraceptive use (binary), as outlined above, were also examined as separate variables, particularly in association with significant predictors of unmet need for contraception.

### Predictors

#### Intimate partner violence

The revised Conflict Tactics Scale (CTS2)^57^ evaluated sexual and physical/injurious IPV dichotomously. This scale comprised three subscales focusing on physical, sexual, and injurious IPV experiences, categorized into lifetime and past 6-month occurrences. This study used past 6-month IPV to examine its more immediate impact on unmet need for contraception. Given that only 19 individuals (14.4%) reported instances of physical or injurious abuse, and considering the similarity between the two subscales' content, these two measures were combined.

#### Substance use

This study assessed past 3-month binge drinking, cannabis use, and other illegal drug use dichotomously using the Risk Behavior Assessment question:^58,59^ “Have you used [the following below] in the past 90 days?” (a) smoked cannabis, (b) other federally illegal substances, including heroin, cocaine, crack, speedball, crystal methamphetamine, nonprescribed stimulants or opiates, ecstasy, or other substances, (c) binge drank (drank four or more alcohol drinks in a period of 6 hours).

#### Mental health diagnoses

Mental health diagnoses were dichotomously scored *via* the query: “Have you ever been told by a doctor that you have [anxiety; bipolar disorder; depression]?”

#### Covariates

This study assessed the effects of relevant covariates from the literature on unmet need for modern contraception, including having a regular gynecologist (yes/no), age (continuous), Black race (dummy coded and compared with other race/ethnicity), Hispanic ethnicity (dummy coded and compared with other race/ethnicity), employment status (unemployed vs. employed), income (less than or greater than $400 a month), high school education (yes/no), relationship/marriage status (yes/no), and insurance, including Medicare or Medicaid (yes/no).

### Statistical analyses

First, descriptive statistics characterized the sample on all predictors and covariates. Second, univariate logistic regression models using generalizing linear modeling (GLM) with robust estimation assessed effects of all covariates on unmet need for modern contraception to determine which were significant. Third, a multivariate logistic regression model using GLM with robust estimation assessed effects of eight main predictors and two significant covariates on unmet need for modern contraception. Due to the small sample size and limited power, only the two covariates that significantly predicted unmet need for modern contraception (age and regular gynecologist) were included in the equation. Finally, chi-square analyses (between two binary predictors), independent *t*-tests (between one binary and one continuous predictor), and bivariate correlations (between two continuous predictors) determined correlations between significant predictors of unmet need for modern contraception with contraceptive type (*i.e*., condoms or other forms of contraception).

## Results

### Descriptive statistics

Among the 132 women studied, 57 women (43.2%) met the criteria for unmet need for modern contraception—indicating that they were at risk of pregnancy while sexually active but did not use contraception despite not intending to conceive or wanting to delay childbirth. Instances of condomless sex in the past 90 days varied widely from 0 to 205 times, with an average of 21.63 instances (standard deviation [SD] = 31.03). Furthermore, 55 women (41.7%) reported using contraception other than condoms.

Descriptive statistics, including IPV occurrences, substance use patterns, mental health diagnoses, and demographic/socioeconomic factors, are provided in Table 1 for the entire sample and subgroups categorized by the presence or absence of unmet need for modern contraception. Within the past 6 months, 14 women (10.6%) reported experiencing sexual IPV, whereas 16 women (12.1%) reported incidents of physical or injurious IPV. Within the past 3 months, 45 women (34.1%) reported binge drinking, 56 (42.4%) reported cannabis use, and 56 (42.4%) reported other illegal drug use. Regarding lifetime diagnoses, 56 women (42.4%) reported anxiety, 77 (58.3%) reported depression, and 49 (37.1%) reported bipolar disorder.

**Table 1.** Descriptive Statistics of Non-pregnancy Seeking Reproductive-Aged Women in New York City Criminal Legal Systems (*N* = 132)

Variable	Sample	Unmet need for modern contraception		*t*-Value		Cohen's d
		No unmet need (*n* = 75; 56.8%)	Unmet need (*n* = 57; 43.2%)			
	M (SD)	M (SD)	M (SD)			
Age, years	34.36 (8.21)	36.15 (8.23)	32.02 (7.62)	2.95	<0.01	0.52

	*n* (%)	*n* (%)	*n* (%)	χ^2^	*p*	Cramer's V
Black race	77 (58.3)	47 (62.7)	30 (52.6)	1.34	0.25	0.10
Hispanic ethnicity	27 (20.5)	14 (18.7)	13 (22.8)	0.34	0.56	0.05
Employed	11 (8.3)	7 (9.3)	4 (7.0)	0.28	0.63	0.04
Monthly income >$400	42 (31.8)	25 (33.3)	17 (29.8)	0.18	0.67	0.04
High school education	81 (61.4)	47 (62.7)	34 (59.6)	0.12	0.72	0.03
In a relationship	105 (79.5)	59 (78.7)	46 (80.7)	0.08	0.77	0.03
Insurance	121 (91.7)	71 (94.7)	50 (87.7)	2.05	0.15	0.13
Regular gynecologist	79 (59.8)	51 (68.0)	28 (49.1)	4.80	0.03	0.19
Sexual IPV (past 6 months)	14 (10.6)	11 (14.7)	3 (5.3)	3.02	0.08	0.15
Physical/injurious IPV (past 6 months)	16 (12.1)	9 (12.0)	7 (12.3)	0.02	0.96	<0.01
Binge drank (past 3 months)	45 (34.1)	27 (36.0)	18 (31.6)	1.30	0.25	0.13
Cannabis (past 3 months)	56 (42.4)	30 (40.0)	26 (45.6)	0.10	0.75	0.03
Other illicit drugs (past 3 months)	56 (42.4)	37 (49.3)	19 (33.3)	3.39	0.07	0.16
Lifetime anxiety	55 (41.7)	26 (34.7)	29 (50.9)	3.50	0.06	0.16
Lifetime depression	77 (58.3)	44 (58.7)	33 (57.9)	<0.01	0.93	<0.01
Lifetime bipolar	49 (37.1)	30 (40.0)	19 (33.3)	0.62	0.43	0.07

IPV, intimate partner violence; SD, standard deviation.

### Generalized linear multivariate logistic regression

The regression equation predicting unmet need for modern contraception from the 10 predictors was significant [χ^2^(10) = 45.21, *p* < 0.01]. See Table 2 for results. Women reporting a diagnosis of an anxiety disorder were nearly 14 times more likely than women without a lifetime diagnosis of an anxiety disorder to have unmet need for modern contraception (odds ratio [OR]: 13.64; 95% confidence interval [CI]: 2.71–68.34; *p* < 0.01), suggesting that they were less likely to use contraception.

**Table 2.** Logistic Regression Estimates of Predictors on Unmet Need for Modern Contraception

Significant predictor	Odds ratio	95% CI	*p*
Regular gynecologist	0.11	0.01–0.86	0.04
Age	0.74	0.63–0.88	<0.01
Physical IPV (past 6 months)	0.14	0.02–1.10	0.06
Sexual IPV (past 6 months)	0.04	0.002–0.86	0.04
Binge drank (past 3 months)	0.14	0.01–2.0	0.15
Cannabis (past 3 months)	2.39	0.42–13.59	0.33
Other illegal drugs (past 3 months)	1.20	0.37–3.93	0.76
Anxiety (lifetime)	13.64	2.71–68.34	<.01
Bipolar disorder (lifetime)	0.06	0.01–0.73	0.03
Depression (lifetime)	2.01	0.40–10.15	0.40

CI, confidence interval.

All other predictors were negatively correlated with unmet need for contraception, suggesting a greater likelihood of using contraception. Women who reported having a regular gynecologist were 89% less likely than those without a regular gynecologist to have unmet need for modern contraception (OR: 0.11; 95% CI: 0.01–0.86; *p* = 0.04). Women older in age were less likely to have unmet need (OR: 0.74; 95% CI: 0.63–0.88; *p* < 0.01). Thus, for every year increase in age, the likelihood of unmet need decreased by 26%. Women reporting past 6-month sexual IPV were 96% less likely than those who did not report sexual IPV to have unmet need (OR: 0.04; 95% CI: 0.002–0.86; *p* = 0.04). Women reporting a lifetime diagnosis of bipolar disorder were 94% less likely than those who did not report a lifetime diagnosis of bipolar disorder to have unmet need (OR: 0.06; 95% CI: 0.05–0.72; *p* = 0.03). No other predictors were significant.

### Associations between significant predictors of unmet need and contraception types

Table 3 illustrates the associations between the significant predictors of unmet need for modern contraception and instances of condomless sex. Table 4 demonstrates the associations between these predictors and contraception other than condoms. Women who had a regular gynecologist had fewer instances of condomless sex (M = 16.49, SD = 23.09) compared with women without one (M = 29.30, SD = 39.08) [*t*(76.41) = 2.15, *p* = 0.04]. Increased age was associated with decreased instances of condomless sex [*r*(132) = −0.23, *p* < 0.01], as well as greater likelihood of using contraception other than condoms (M_age_ = 36.80, SD = 7.69, for women with a regular gynecologist; M_age_ = 33.06, SD = 7.87, for women without a regular gynecologist) [*t*(117.27) = −2.67, *p* < 0.01]. Bipolar disorder was associated with increased likelihood of using contraception other than condoms [χ^2^(1) = 3.91, *p* = 0.05; Cramer's *V* = 0.18].

**Table 3.** Bivariate Associations of Significant Predictors with Individual Instances of Condomless Sex

Variable	Instances of condomless sex among women who did not endorse predictor variable, M (SD)	Instances of condomless sex among women who endorsed predictor variable, M (SD)	*t* *-Value*	*p*	*Cohen's *d
Regular gynecologist	29.30 (39.08)	16.49 (23.09)	2.15	0.04	0.42
Sexual IPV (past 6 months)	21.22 (31.01)	24.21 (32.28)	−0.33	0.74	−0.09
Lifetime anxiety disorder	20.19 (28.15)	23.65 (34.85)	−0.63	0.54	−0.11
Lifetime bipolar disorder	20.57 (27.83)	23.45 (36.05)	−0.51	0.61	−0.09

			*R*-value	*p*	
Age			−0.227	<0.01	

**Table 4.** Bivariate Associations of Significant Predictors with Other Forms of Contraception

Variable	*n (%) of Women using other contraception among women who did not endorse this variable*	*n (%) of Women using other contraception among women who endorsed this variable*	χ^2^-Value	*p*	Cramer's V
Regular gynecologist	17 (34.0)	38 (76.0)	3.38	0.07	0.16
Sexual IPV (past 6 months)	47 (41.6)	8 (66.7)	2.77	0.18	0.15
Lifetime anxiety	35 (48.6)	20 (37.7)	1.47	0.23	0.11
Lifetime bipolar disorder	29 (37.2)	26 (55.3)	3.92	0.05	0.18

	Age of women not using other contraception, M (SD)	Age of women using other contraception, M (SD)	*t* *-Value*	*p*	*Cohen's *d
Age	33.06 (7.87)	36.80 (7.69)	−0.23	<0.01	−0.48

## Discussion

This study examined associations between interpersonal- and individual-level factors (*i.e*., IPV, mental disorders, substance use) associated with unmet need for modern contraception among 132 non-pregnancy seeking reproductive-aged WICL. The analysis accounted for significant demographic and socioeconomic covariates, including women's age and access to a regular gynecologist. Younger women, without a regular gynecologist, reporting lifetime diagnoses of anxiety disorders were more likely to experience unmet need for modern contraception. Specifically, younger women had greater instances of condomless sex and were also less inclined to use alternative forms of contraception. In addition, women without a regular gynecologist had greater instances of condomless sex.

Alternatively, women reporting lifetime diagnoses of bipolar disorder and endorsing histories of sexual IPV were less likely to meet the criteria for unmet need for contraception. Specifically, women with bipolar disorder were more likely to use contraceptives other than condoms. These findings highlight the opportunity within the CL system to provide integrated health services catering to the overlapping needs identified in these factors, as elaborated below.

### Access to a regular gynecologist

Regarding socioeconomic factors, research has found that women who are unemployed, with lower income and without insurance or on public health insurance have had higher rates of unmet need for contraception.^27,60–62^ Nevertheless, in more homogeneous groups such as WICL, where factors such as unemployment and limited insurance prevail, distinctions based on these factors may be more restricted. In this specific sample, having a regular gynecologist (endorsed by 59.8% of women) better differentiated unmet need than employment, income, or insurance. Specifically, there was a significant relationship between having a gynecologist and a lower probability of engaging in condomless sex, suggesting that SRH providers play a role in informing women about contraceptive choices. Although not a significant predictor for alternative contraception use in this sample (*p* = 0.07), further exploration in a larger study could yield significant results.

Establishing a connection with SRH providers during involvement in the CL system is vital. Studies indicate that access to onsite contraceptive services enhances the likelihood of women initiating usage compared with mere community referrals.^48^ For example, New York's prison system, during 2009–2013, collaborated with Planned Parenthood to provide prerelease contraceptive services.^63^ Contraceptive services designed for WICL should prioritize personalized, patient-centered SRH counseling. This approach involves a holistic conversation about women's reproductive life objectives rather than solely focusing on contraception.^48^ Health care providers should be especially sensitive to the historical impact of eugenics, which forcefully restricted reproduction in communities of color. Providers should offer information and access to varied methods while considering patients' goals and needs without undue emphasis on specific forms of contraception.^5,64,65^

### Age

This study found younger age to be a predictor of unmet need for modern contraception among 18- to 44-year-old women, consistent with the literature.^27,30^ Younger women were more likely to have condomless sex and less likely to use other forms of contraception than older women within this age range. Thus, targeted efforts, including improved assessments, prevention methods, education, and policies for younger age brackets within the broader “reproductive-aged” category, would be beneficial. More nuanced questioning to accurately gauge pregnancy intentions among younger women, who may experience more uncertainty, may be needed to discern pregnancy intentions effectively.

Previous research utilized Likert scale measurements (*e.g*., answering 1–5 on the item “I would be very upset if I were pregnant right now”)^66^ and specific queries (*e.g*., “If you are pregnant now, what are your plans for the pregnancy? abortion, adoption, parenting, or uncertainty”).^66^ Notably, the literature indicates that younger women tend to use less effective pregnancy prevention methods such as birth control pills or condoms rather than IUDs.^66^ Therefore, short-term holding facilities should enable women to maintain their current contraception methods to avoid disruptions and reduce the heightened risk of unintended pregnancy upon their return to the community.^66^

### Intimate partner violence

Although this study investigated IPV as a barrier to contraceptive use, it found an inverse association between sexual IPV and unmet need for modern contraception. This finding aligns with certain research findings,^38,53,67,68^ but contradicts others.^37,52,69^ This link might be tied to the higher probability of women experiencing reproductive coercion,^42^ leading them to be more likely to use certain contraceptive methods, as IUDs or oral contraceptive pills, which they can conceal from their partners.^38,40,50,70,71^ While the association between sexual IPV and alternative contraceptives was not statistically significant in this study (*p* = 0.10), a more robust study might uncover this relationship.

To support women experiencing reproductive coercion, the American College of Obstetricians and Gynecologists (ACOG) suggests that SRH providers offer long-acting reversible contraception options. Therefore, integrating these choices within CL settings, alongside other options, could be particularly beneficial. Studies indicate that providing IUDs and implants within CL settings is both safe and feasible in the context of integrated SRH care.^72^ For women opting for an IUD, maintaining separate appointments for counseling and insertion is crucial to ensure that they have ample time to make informed decisions about their contraceptive choices.^48^

It is also crucial to note that among women facing sexual IPV, increased rates of contraceptive use in some research have not been linked to reduced risk of unintended pregnancies. This discrepancy could be due to higher rates of contraceptive failure, implying that women experiencing IPV might need additional support to maintain effective contraceptive use.^38^ Future research should aim to elucidate potential variations in associations between physical and sexual IPV and their impact on unmet need for contraception.

### Mental disorders

In this study, a reported lifetime diagnosis of an anxiety disorder was the strongest predictor of unmet need for modern contraception, increasing its likelihood nearly 14-fold. As this study is cross-sectional, a causal relationship cannot be determined. Nevertheless, possible explanations are that women with anxiety disorders may be more likely to perceive somatic side effects of hormonal contraception and discontinue it more readily.^73^ Women with anxiety disorders may have lower self-efficacy for condom use, or impaired judgment and risk evaluation, increasing their likelihood of engaging in condomless sex.^74–77^ Women who have greater life stressors causing anxiety may experience more barriers to accessing and utilizing contraception, although this relationship may also exist with other mental disorders. Less research has focused on individual effects of anxiety disorders on contraceptive outcomes compared with other disorders, and these findings underscore the importance of more exploration.

Contrarily, a lifetime diagnosis of bipolar disorder in this study was associated with a decreased likelihood of unmet need for modern contraception. Specifically, women reporting a lifetime diagnosis of bipolar disorder were more inclined to use contraceptives other than condoms. Limited research has specifically delved into the influence of bipolar disorder on contraceptive use, distinct from other mental disorders or serious mental illness (SMI), which includes severe impairments such as major depressive disorder, schizophrenia, and schizoaffective disorder.^78^ While research has indicated greater rates of unintended pregnancy and reproductive concerns among women with bipolar disorder,^79,80^ studies have varied in regard to reported differences of contraceptive use.

Moreover, similar to our study, some research has indicated higher contraceptive use among women with bipolar disorder, although with preferences for more “traditional” methods, including withdrawal (which was not included in this current study under modern contraceptive methods).^81^ These findings indicate the utility of querying contraceptive use in more detail, as a dichotomous assessment of contraceptive use does not capture consistency of use, which may be particular concerns among this population. Furthermore, the findings of this study indicate a need for research to better understand the experiences of WICL with bipolar disorder, particularly women taking medication, as they might experience compounded marginalization, and potential risks for coercion by health care providers in their family planning decisions.

### Substance use

Finally, in this study, there were no significant associations between past 3-month substance use of any kind and unmet need for modern contraception. Most studies exploring substance use and contraceptive practices have concentrated on groups with SUDs,^3,54,82^ which might better predict contraceptive outcomes and unintended pregnancies. Moreover, this sample might not exhibit enough diversity in distinguishing various profiles among women who use substances, as all participants were required to report recent substance use to join the initial study. Furthermore, WICL who acknowledge drug use may be more inclined to receive substance use treatment, potentially aiding in connecting them to various forms of care, including contraceptive services. Collaborative initiatives targeting both substance use and SRH among reproductive-aged women can reduce barriers to care for women dealing with SUDs.^23^

### Limitations

While these findings offer valuable insights, there are several limitations that impact interpretation. Our determination of pregnancy risk relied on an age range criterion of 18–44 years, yet not all women within this bracket might be capable of conceiving, and those outside this range might still face pregnancy risks. In addition, part of our definition of other forms of contraception might not encompass all women at risk of unintended pregnancy as categorization was assessed dichotomously and assumed ongoing usage during sexual encounters.

Furthermore, except for age, all predictor variables were evaluated dichotomously, potentially overlooking important variance. Moreover, our evaluation of mental health diagnoses does not provide a comprehensive representation of women's symptoms, which may have resulted in higher estimates of potential diagnoses than would be expected. Our sample size was relatively small, impacting our ability to detect significant effects. Finally, variations in incarceration systems across the United States and disparities in accessing health care within these systems pose challenges in generalizing this information to the wider US population.

## Conclusions

This study contributes valuable insights to the growing body of research on SRH among WICL by examining associations of IPV, mental health diagnoses, and substance use with contraceptive methods other than condoms, while considering women's pregnancy risk and intentions. Findings revealed unique impacts of distinct mental health disorders and types of IPV. These results underscore an opportunity within CL systems to deliver comprehensive health services that address the intersecting needs of this population. However, these complex findings also emphasize the need for further research and a more nuanced comprehension of these relationships for the development of more effective assessment and intervention strategies to address unmet need for modern contraception among WICL.

## Abbreviations Used

CI: confidence interval
CL: criminal legal
GLM: generalizing linear modeling
IPV: intimate partner violence
IUD: intrauterine device
OR: odds ratio
SD: standard deviation
SRH: sexual and reproductive health
SUD: substance use disorder
WICL: women involved in criminal legal systems
WORTH: Women on the Road to Health
